# Immunochemical Determination of IgE and IgG Autoantibodies in Patients with Chronic Spontaneous Urticaria: A Narrative Review

**DOI:** 10.3390/antib15030044

**Published:** 2026-05-28

**Authors:** Chrysoula-Evangelia Karachaliou, Evangelia Livaniou

**Affiliations:** Immunopeptide Chemistry Laboratory, Institute of Nuclear & Radiological Sciences & Technology, Energy and Safety (INRASTES), National Centre for Scientific Research “Demokritos” (NCSRD), Agia Paraskevi 15310, Greece; xrisak15@hotmail.com

**Keywords:** chronic spontaneous urticaria (CSU), autoimmune type I CSU, autoimmune type IIb CSU, specific IgE autoantibodies, specific IgG autoantibodies, total IgE, autoantigen, autoallergen, immunochemical assay, ELISA

## Abstract

**Background/Objectives**: Chronic spontaneous urticaria (CSU) is characterized by almost daily wheals or angioedema lasting for more than six weeks and not attributable to a defined inducing factor. CSU reportedly affects 1–2% of the general population and may lead to a substantial impairment in patients’ quality of life. Thus, developing methods that enable early diagnosis and assessment of disease activity is a major objective for scientists and clinicians. **Methods**: A significant proportion of CSU cases appears to be associated with autoimmune mechanisms, which mainly involve IgE autoantibodies (type I CSU), IgG autoantibodies (type IIb CSU), or both (type I and type IIb overlap). To this end, detection of specific IgE and/or IgG autoantibodies in CSU patients using biological or immunochemical assays can offer valuable information and enable further investigation and better management of the disease. **Results**: This review focuses on and presents various immunochemical assays, mainly ELISAs, for determining specific IgE and/or IgG autoantibodies, along with immunochemical methods for quantifying total IgE levels as an additional biomarker in CSU patients; the development and/or application of these assays has been reported in several papers published in the last decade on CSU. **Conclusions**: The methods presented have recently been applied and have substantially contributed to CSU diagnosis, endotyping and prediction of response to various treatments. Further validation of the existing immunochemical assays along with the development of reliable assays for novel autoantibodies and/or autoantigens will deepen our understanding of CSU pathogenesis and support the clinical diagnosis and treatment of CSU.

## 1. Scope of the Review

The main scope of this review article is to present information concerning various immunochemical assays that have been used for CSU diagnosis/prognosis/response to therapy. Information has been extracted from articles that clearly referred to and/or described in detail the immunochemical assays, especially in-house developed ones, used to determine specific IgE autoantibodies, IgG autoantibodies and/or total IgE (tIgE) in CSU patients. These articles have been selected from a broader pool of articles we generated by searching the PubMed database (period 2019–2025) using various search terms, alone or in combination, such as CSU, autoimmune CSU, type I CSU, type IIb CSU, biomarkers, IgE autoantibodies, autoallergen, IgG autoantibodies, tIgE, immunoassay, and immunochemical assay.

## 2. Introduction

### 2.1. Autoimmune Diseases

Autoimmune disease (AD) is a term used to describe various disorders characterized by a broad range of clinical symptoms. All ADs have in common the improper activation and abnormal function of the immune system; this dysfunction leads to an attack of the patients’ own molecules, cells, tissues, and organs by the improperly activated immune system, which may result in prolonged inflammation and tissue/organ damage. It is estimated that ADs affect a large number of individuals, reaching 5–7% of the global population, and their prevalence will probably increase in the near future, mainly in industrialized countries. The pathogenesis of ADs is mostly unknown, although various genetic, epigenetic, and environmental factors have been implicated as putative causes; as has also been well documented, specific viral infections may lead to the development of ADs [1,2,3].

The development of ADs is closely associated with the presence of autoantibodies that recognize specific self-antigens; thus, the determination of specific autoantibodies in patients is considered an excellent analytical tool for diagnosing ADs. Moreover, determination of autoantibodies may offer valuable information on the disease prediction and prognosis and help assess patients’ potential response to disease treatment(s) [2,4,5].

### 2.2. Chronic Spontaneous Urticaria

According to the 2022 guidelines of the European Academy of Allergy and Clinical Immunology (EAACI), urticaria is a skin disorder characterized by the development of wheals, angioedema, or both [6]. Urticaria has a high disease burden and significantly affects patients’ quality of life, often leading to high health care costs. Urticaria can be classified as chronic or acute. Chronic urticaria (CU) is characterized by daily (or almost daily) wheals and/or angioedema lasting for at least six weeks; on the other hand, in acute urticaria (AU), wheals or angioedema last for less than six weeks [7].

Chronic spontaneous urticaria (CSU) and chronic inducible urticaria (CIndU) are two subgroups of CU. CIndU is induced by a specific stimulus, e.g., cold or pressure [7,8]; for instance, a 2023 case report described a patient with progesterone-induced CU, which was probably triggered by an intrauterine device implant delivering levonorgestrel [9]. On the other hand, CSU refers to CU cases that cannot be attributed to a defined inducing factor [7]. As reported, CSU affects 1–2% of the global population, with a higher prevalence among females and people older than 20 years [10,11].

Mast cells and basophils are considered key effector cells in CSU, although eosinophils may also be important in CSU pathogenesis. Abnormal activation of these effector cells is thought to trigger the release of proinflammatory factors, e.g., histamine, thus resulting in disease development [7,12,13].

The underlying causes of CSU are not yet completely elucidated, but autoimmune mechanisms seem to be involved in a substantial proportion of CSU cases. For instance, autoantibodies are present in 30% to 40% of CSU patients, which is indicative of an autoimmune background; interestingly, both IgG- and IgE autoantibodies seem to be involved in the mechanisms underlying autoimmune CSU [6,7,10,12,13,14].

Atopic and autoimmune (along with psychiatric) diseases are prevalent comorbidities in patients with CSU [15]. Notably, CSU has frequently been associated with autoimmune thyroid diseases (mainly Hashimoto’s thyroiditis) and vitiligo, type 1 diabetes mellitus, systemic lupus erythematosus, and rheumatoid arthritis, which further supports an autoimmune origin of many CSU cases; moreover, a series of recent findings further support the suggestion that autoimmune mechanisms often underlie CSU [7,11,16,17].

### 2.3. Endotypes of CSU

CSU cases manifest in several forms, i.e., as type I, type IIb, combined type I/IIb, and variants that do not fit either category. Type I, type IIb, and combined type I/IIb are considered forms of autoimmune CSU [18].

In autoimmune CSU, effector mast cells can be activated through the generation of complexes between IgE and IgG autoantibodies to the high-affinity IgE receptors (FcεRI receptors) [18]. In addition, B cell receptor signaling might be involved in autoimmune CSU through the generation of autoreactive B cells and production of autoantibodies; in both FcεRI and B-cell receptor signaling pathways, Bruton tyrosine kinase (BTK) seems to play a key role [19]. Moreover, recent findings implicate complement and mas-related G protein-coupled receptor X2 (MRGPRX2) in autoimmune-related mast cell activation associated with CSU [12,16].

Type I CSU (also known as “autoallergic”), which is associated with IgE autoantibodies against various autoantigens (“autoallergens”), and type IIb CSU, which is associated with IgG autoantibodies, principally against IgE or FcεRI, have been well characterized [16]. Type I CSU appears to be more prevalent among patients with autoimmune CSU, as specific research reports have indicated that a high proportion (between 38% and 58%) of CSU patients may be assigned to endotype I [18]. Patients with type I CSU exhibit less severe clinical symptoms. On the other hand, patients (mostly female) who exhibit characteristics of type IIb CSU have been associated with prolonged disease duration, more severe CSU activity, concomitant autoimmune diseases, such as thyroiditis and vitiligo, lower quality of life, and resistance to standard treatment options; type IIb CSU patients were the first reported to have autoimmune CSU [12,16].

As already mentioned, a subpopulation of CSU patients has an overlap of the two endotypes associated with autoimmunity [16]. More specifically, most CSU patients present signs of endotype I, which may or may not coexist with signs of endotype IIb [20]; on the other hand, as reported in recent studies, type IIb patients frequently present coexisting type I characteristics (while the vice versa is uncommon). Some researchers have suggested that type I CSU might develop into type IIb CSU, as patients may also produce IgG autoantibodies over time [18,20]; nevertheless, further research is needed to confirm this suggestion [21].

CSU variants that cannot be defined as type I, type IIb, or combined type I/IIb, may be considered as non-autoimmune and/or cases of unknown origin. Although some information can be found in the literature, the exact proportion of patients with type I CSU, type IIb CSU, both, or non-autoimmune/non-classified CSU is mostly unknown.

Further details concerning the types of autoimmune CSU are presented below.

#### 2.3.1. Endotype I (Autoallergic)

In type I autoimmune CSU (autoallergic CSU), the effector cells are activated when an autoallergen forms a complex with IgE [19,22]. As in classical allergy, mast cells and basophils are activated through the cross-linking of FcεRI-bound IgE, which may consequently lead to inflammation and various clinical manifestations including wheals [23,24]. In addition to IgE-dependent pathways, IgE-independent mast cell activation has also been reported, e.g., through complement and MRGPRX2 receptor [12].

Major autoallergens include IL24, tissue factor, tissue transglutaminase, thyroglobulin (TG), and thyroid peroxidase (TPO) [19,22] and it should be noted that TPO was the first self-antigen/autoallergen to be identified [16]. Additional autoallergens have also been identified, including double-stranded DNA and various other compounds [18,23]. FcεRI may itself be an autoallergen, as variably increased levels of anti-FcεRI-IgE have been detected in patients with CSU [19]. To date, researchers have reported approximately 200–250 self-molecules that may act as autoallergens in CSU [16]. As reported, most patients with endotype I CSU have IgE autoantibodies against one specific autoallergen, while fewer patients have IgE autoantibodies against more than one autoallergen, e.g., against both, TPO and IL24 [21].

Diagnosis of type I CSU: This may be achieved through testing for specific IgE autoantibodies, mainly with ELISA and basophil activation tests [23]. An additional biomarker is the tIgE level, which is usually high (typically >100 IU/mL, or normal (typically >43 IU/mL) [16,18]. Given that assay kits for determining specific IgE autoantibodies are not widely available, tIgE levels may practically be considered the primary biomarker of type I CSU; it should be noted, however, that tIgE may be affected by various factors, including exogenously induced allergic conditions, while the correlation between tIgE and specific IgE autoantibodies is not always strong [16,18].

#### 2.3.2. Endotype IIb

Type IIb autoimmune CSU is predominantly mediated by IgG autoantibodies targeting either the FcεRI or IgE molecules already bound to FcεRI [18]; IgG autoantibodies directed at other mast-cell-activating receptors, such as MRGPRX2 and C5aR, might also play a role in type IIb CSU [16]. Moreover, patients with type IIb CSU are often found to have anti-TPO-IgG autoantibodies [16,22]. IgG1 and IgG3 are considered the main IgG subclasses that participate in mast cell triggering and complement activation, while IgG4 seem to play a less important role in the activation process [16,18]. Other features associated with type IIb CSU include low levels of IgE (typically <43 IU/mL), low levels of IgA, basopenia, and eosinopenia [16,22].

As reported, type IIb autoimmune CSU is present in less than 10% of CSU patients when the criteria presented in the international Profiling Urticaria for the Identification of Subtypes (PURIST) study are applied to endotyping [16,22].

Diagnosis of type IIb CSU: As reported, the ‘‘criterion standard’’ for the diagnosis of type IIb CSU (as described in the above-mentioned PURIST study) is (i) a positive autologous serum skin test (ASST, described in Section 2.5), (ii) a positive basophil histamine release assay or a basophil activation test (BHRA or BAT, described in Section 2.5), and (iii) detection of anti-FcεRI-IgG, anti-IgE-IgG, or both, using immunoassay [13,18,22,25]. As immunoassays for anti-IgE-IgG and/or anti-FcεRI-IgG may not be widely available for routine clinical application, an alternative diagnostic marker is the combination of high anti-TPO-IgG and low tIgE levels [16,18,22,26]. Thus, a new marker of type IIb is the high ratio of anti-TPO-IgG to tIgE (typical cut-off value: 2.88) [21].

#### 2.3.3. Type I and Type IIb Overlap

As already mentioned, it is highly possible that patients with autoimmune CSU cannot be strictly subdivided into either type I or type IIb forms of the disease, based on the presence of either IgE or IgG autoantibodies against self-antigens, respectively. However, there is increasing evidence that autoantibodies of both classes, IgE and IgG, may co-exist and can thus be detected in the same patients; such patients are characterized by an overlap of type I and type IIb autoimmune mechanisms and disease features, while they may exhibit a distinct response pattern to CSU treatments [16,22].

### 2.4. CSU Therapeutics

Only a small number of therapies are approved for CSU treatment, and patients can be refractory to these treatments [27]. In efforts to develop more effective therapies and improve patients’ quality of life, a shift from conventional antihistamines to biologics has recently been reported [28]. More specifically, standard therapy for CSU includes second-generation antihistamines and the humanized, anti-IgE monoclonal antibody omalizumab. However, many patients tend to be refractory to these therapies [27]. Current management guidelines recommend step-up administration of second-generation H1-antihistamines to fourfold the approved dose (first line), followed by omalizumab (second line) and cyclosporin or other immunosuppressants (third line). However, in many patients, chronic urticaria does not respond to this linear approach due to heterogeneous underlying mechanisms, while novel therapies have emerged and are under investigation in clinical trials [8,18].

As reported, less than 10% of patients have complete control of their CSU with second-generation H1-antihistamines, i.e., the first-line treatment, while about 70% of patients with antihistamine-refractory CSU do not reach complete control with omalizumab, either [29].

In addition to omalizumab, other types of anti-IgE therapies have been suggested, e.g., ligelizumab, which has undergone phase 3 clinical trials [10].

Interestingly, endotype classification has been shown to predict therapeutic response [12,22]. Thus, type I CSU generally shows a better response to up-dosed second-generation antihistamines and a good response to omalizumab, if necessary. On the other hand, type IIb CSU often needs immunosuppressive drugs [12,13,22]. Thus, personalized therapeutic approaches predominantly based on endotype/biomarker characteristics may be followed in the near future [8,18].

Novel therapies are needed to treat CSU, especially for patients with poor or late response to the currently widely used therapeutic options, such as treatment with omalizumab [29]. Among the novel treatments reported, BTK inhibitors, e.g., remibrutinib and rilzabrutinib, which block IgE-mediated histamine release from mast cells; anti-tyrosine protein kinase (anti-KIT) monoclonal antibodies, e.g., barzolvolimab; anti-cytokine therapies, e.g., dupilumab; and inhibitors of tumor necrosis factor alpha (TNFα) have been successfully applied in some cases. Furthermore, various other novel drugs have been suggested as potential treatments for CSU, some of which are currently under investigation in clinical trials [27,29].

### 2.5. Functional/Biological Assays for CSU Diagnosis/Prognosis/Response to Therapy: ASST, BAT, BHRA

A well-established method for autoimmune CSU diagnosis is the autologous serum skin test (ASST). In this test, a sample of the patient’s own serum is intradermally injected into his/her skin. The development of a reactive wheal at the injection site indicates a positive result [18]. Interestingly, positive ASST may be linked to circulating histamine releasing factors other than autoantibodies, e.g., coagulation factors or MRGPRX2 agonists [22]. A significant association has recently been shown between ASST positivity and disease severity, particularly in type IIb CSU [30]. Although theoretically a useful tool to detect autoreactivity, ASST has been associated with a series of limitations, which practically limit its routine clinical utility [18].

Two other well-recognized laboratory assays are the basophil histamine release assay (BHRA) and the basophil activation test (BAT). BHRA measures the histamine released from donor basophils after exposure to the patient’s serum. As reported, the clinical utility of BHRA is limited due to the variable basophil reactivity among individual donors. The assay’s laborious protocol and the need for freshly prepared basophils from donors’ blood are additional obstacles preventing wide application [18]. BAT, which is based on flow cytometry measurements, provides a more straightforward approach to studying basophil function. This method monitors the expression of certain surface proteins—mainly CD63 and CD203c—on basophil membranes when the latter are activated. Direct and indirect BAT protocols have been reported in the literature. Typically, a positive BAT result is defined as the detection of CD63 on more than 5% of basophils in response to the patient’s serum. When compared with BHRA, BAT is thought to offer greater reliability and precision. It should be noted that discrepancies may sometimes be observed between BAT and BHRA results, the origins of which remain to be further investigated and explained [18,23]. A systematic literature review assessing the relationship between BHRA/BAT positivity and clinical features/laboratory parameters of CSU patients was published in 2023 [31].

## 3. Binding-Based Immunochemical Assays for CSU Diagnosis/Prognosis/Response to Therapy: Determination of Specific IgE Autoantibodies, Specific IgG Autoantibodies, and/or Total IgE Levels

### 3.1. Measurement of IgE Autoantibodies Against Specific Autoantigens and/or Total IgE Levels in CSU Patients

A number of articles referring to the immunochemical-assay-based measurement of IgE autoantibodies against specific autoantigens, sometimes along with measurement of total IgE levels, in CSU patients have appeared in recent literature; these articles are detailed below.

In a 2023 article, the authors measured the levels of IgE against the autoantigen transglutaminase 2 (anti-Tg2-IgE) in CSU patients and examined the correlation between anti-Tg2-IgE with the clinical and serological features of CSU. An in-house-developed IgE-capture ELISA was used to quantify anti-Tg2-IgE levels in 160 CSU patients and 54 healthy control individuals. Purified chimeric human anti-TPO-IgE and purified human TPO from thyroid glands were used to set up a standard curve as previously described [32]. CSU patients with high and low serum levels of anti-Tg2-IgE were examined for differences in their clinical features and laboratory markers, including tIgE, along with total IgG (tIgG), total IgA (tIgA), and total IgM (tIgM). The results showed that a distinct and significant proportion of CSU patients showed elevated anti-Tg2-IgE levels, which supports the further investigation of the role of anti-Tg2-IgE and Tg2 in the pathogenesis of CSU [33].

The association of thyroid autoantibodies with allergic diseases was assessed in an article published in 2022. The results showed that thyroid autoantibodies, presumably of the IgE class, such as autoantibodies against thyroglobulin (anti-TG) and thyroid peroxidase (anti-TPO), and especially anti-TG autoantibodies, are significantly associated with allergic disorders, such as atopic rhinitis, atopic dermatitis and CSU; it might therefore be clinically useful to measure their levels and correlate them with the clinical features and the disease course in allergic patients [34].

In an article published in 2021, the authors investigated the IgE response against the eosinophil proteins eosinophil peroxidase (EPX) and eosinophil cationic protein (ECP) in patients with CSU and atopic dermatitis as well as in control individuals by means of in-house-developed ELISAs; anti-TPO-IgE was also measured in all groups using an in-house-developed ELISA. IgE autoantibodies against EPX and ECP were present in both groups of patients. In the CSU group, higher anti-TPO-IgE levels were measured, while a correlation was observed between the anti-EPX-IgE and anti-TPO-IgE levels. Putative cross-reactivity between thyroid and eosinophil proteins was assessed with ELISA and immunoblotting IgE-binding inhibition assays using an appropriately modified version of a previous protocol [35]; the cross-reactivity that was observed between thyroid and EPX proteins might explain the frequent comorbidity between autoimmune thyroid disease and CSU. Total IgE levels were also measured in all groups; tIgE levels were similar in the CSU and control groups, while the atopic dermatitis group had higher levels of tIgE. Overall, based on the results presented, the role of IgE autoantibodies against eosinophilic proteins in the pathogenesis of CSU (and atopic dermatitis) deserves to be further evaluated [36].

In another article published in 2021, a group of patients with CSU were investigated for their anti-TPO-IgE, tIgE and tIgA levels, along with tIgM and tIgG levels; anti-TPO-IgE was measured with an in-house-developed ELISA. The results showed that patients with elevated anti-TPO-IgE levels had lower tIgA and tIgE levels. Overall, the findings of this study support screening of CSU patients for their serum tIgA and tIgE, to further assess the role of the latter immunoglobulins as putative disease biomarkers [37].

In a previous article published in 2019, the clinical profile of CSU patients was correlated with serum anti-TPO-IgE autoantibodies. The anti-TPO-IgE levels were determined with an in-house-developed ELISA, while tIgE was also measured. As reported, patients with anti-TPO-IgE positivity had different clinical features and higher tIgE levels compared with patients without anti-TPO-IgE. Although increased levels of tIgE were measured in patients with anti-TPO-IgE, a rather weak correlation between tIgE and anti-TPO-IgE was found. Overall, according to the authors, anti-TPO-IgE is a useful biomarker for differentiating among different CSU phenotypes, while the increase in anti-TPO-IgE levels during disease exacerbations seems to support an association of this autoantibody with the pathogenesis of CSU [38].

Details concerning the measurement of specific IgE autoantibodies and/or tIgE levels in CSU patients are summarized in Table 1.

### 3.2. Measurement of Specific IgG Autoantibodies and/or Total IgE Levels in CSU Patients

Many articles referring to the immunochemical-assay-based measurement of IgG antibodies against specific autoantigens (along with specific IgM and IgA antibodies in some cases) have appeared in the recent literature; in most of these, total IgE levels were also measured. The articles are detailed below.

In a recently published article, the authors assessed anti-IgE-IgG and anti-FcεRIα-IgG autoantibodies along with tIgE and free IgE (fIgE) in 61 patients with CSU before omalizumab administration, to study whether the IgG autoantibodies’ levels could be applied to predict response to omalizumab treatment. Anti-IgE-IgG, anti-FcεRIα-IgG, tIgE, and fIgE were measured in human sera using ELISAs. The study showed that high levels of serum anti-IgE-IgG autoantibodies indicate non-response during the early phase of omalizumab administration. On the other hand, the anti-IgE-IgG autoantibody levels in responders and non-responders did not significantly differ in the late phase of treatment. Moreover, high levels of serum anti-FcεRIα-IgG autoantibodies indicated non-response at the early phase of omalizumab administration [40].

In another recent study, the authors evaluated the prevalence of antinuclear antibodies (ANA) in 60 patients with CSU. ANAs, predominantly of the IgG class, were detected via indirect immunofluorescence using the HEp-20-10 biochip kit, and 37 of the CSU patients were ANA positive. Total IgE was also measured and a decrease in tIgE levels among ANA-positive patients was observed compared with ANA-negative ones, which may reflect endotype IIb autoimmunity [41].

A recent publication reported hypothyroidism along with significantly higher occurrence of anti-TPO-IgG autoantibodies in CSU patients versus control individuals, as assessed with commercially available immunoassays, which agrees with results of previous relevant studies. On the other hand, TPO expression was not detected in skin biopsies from both CSU patients and control subjects. Thus, according to the authors, anti-TPO-IgG autoantibodies and thyroid dysfunction are correlated with CSU, but TPO itself may not cause a direct effect on the cutaneous manifestations of the disease [42].

In a recently published retrospective study, the researchers investigated whether the eosinophil/lymphocyte, neutrophil/lymphocyte, platelet/lymphocyte, and basophil/lymphocyte ratios (ELR, NLR, PLR, and BLR, respectively) may predict the response to omalizumab therapeutic administration to CSU patients. To this end, various parameters were determined before, after three, and after six months of treatment, including anti-TPO-IgG and tIgE levels. Anti-TPO-IgG autoantibodies and tIgE levels were measured with immunoassays. According to the findings, no significant changes were observed in NLR, ELR, PLR, and BLR values during a six-month period of omalizumab administration. A comparison between endotype I and endotype IIb CSU revealed that eosinophils, basophils, BLR, and tIgE were significantly higher in endotype I CSU, while anti-TPO-IgG and the anti-TPO-IgG/tIgE ratio were significantly lower in endotype I; moreover, the initial values of ELR and BLR were significantly associated with the initial tIgE levels and anti-TPO-IgG/tIgE ratio [43].

In a recent article, concentration and avidity of anti-FcεRIα-IgG and anti-IgE-IgG autoantibodies were evaluated in a group of CSU patients. Evaluations were performed using in-house-developed ELISAs at baseline and six months later. The authors observed an increase in the quantity of anti-FcεRI-IgG and anti-IgE-IgG autoantibodies along with a simultaneous decrease in avidity that was observed in all CSU patients after a 6-month period. According to the authors, the quantity and avidity of anti-FcεRI-IgG and anti-IgE-IgG autoantibodies may change over time and do not correlate with disease activity; thus, these autoantibodies cannot be considered reliable biomarkers for the diagnosis of autoimmune CSU, while the BAT can be considered more suitable for diagnosing autoimmune CSU [44].

In a recently published retrospective study, various parameters determined in CSU patients before omalizumab treatment were investigated as putative predictors of response to omalizumab administration. Total IgE, anti-TPO-IgG, and anti-TPO-IgG/tIgE ratio, all of which had been determined as described in a previous study [45], were among the parameters investigated. In addition, anti-FcεRIα-IgG autoantibodies were investigated as a putative predictor, which had been detected with a biological assay (BHRA). The validated Outcome and Assessment Information Set Database (OASIS-D) rating system was used to assess responsiveness to omalizumab. The study showed that high levels of anti-TPO-IgG were significantly associated with a poor response to omalizumab. However, BHRA, tIgE, as well as anti-TPO-IgG/tIgE ratios were not predictive of the patients’ response to omalizumab administration. Overall, the study confirms previous findings that high levels of anti-TPO-IgG autoantibodies can serve as a reliable predictor of poor response to omalizumab therapy [46].

In another recently published retrospective study, disease severity, comorbidity, and serum levels of tIgE and anti-thyroid autoantibodies, namely anti-TPO-IgG, were evaluated in CSU patients receiving long-term omalizumab treatment (up to eight years). Anti-TPO-IgG and tIgE levels were determined with enzyme fluoroimmunoassays. The authors reported that normal tIgE levels and thyroid autoimmunity were correlated with a higher risk for discontinuation of omalizumab due to treatment inefficacy [47].

In a recently published case report, the authors presented a patient with chronic idiopathic urticaria who did not respond to omalizumab treatment, but she showed a rapid response to the BTK inhibitor acalabrutinib. The patient had a high proportion of CD203c-positive basophils and strong positivity for anti-FcεRI-IgG/anti-IgE-IgG autoantibodies, as detected using a functionality-based assay (the FIERA test) [48].

In a study published in 2023, the researchers investigated whether anti-HSP10-IgG autoantibodies are present in patients with CSU. To address this aim, they used a human proteome microarray and found that six potential autoantibodies showed higher levels in 10 CSU patients compared with 10 control subjects, including the anti-HSP10-IgG autoantibody. The latter was further quantified by immune dot-blot assay in the sera of 86 CSU patients and 44 control subjects. The results showed that the CSU patients had higher anti-HSP10-IgG positivity and lower serum HSP10 levels when compared with the control group, while disease severity was associated with anti-HSP10-IgG positivity. Total IgE was also measured using an immunoassay system and was not correlated with the baseline serum HSP10 levels [49].

In a case study reported in 2023, the authors described a young woman with myasthenia gravis who presented with clinical symptoms of CSU. The patient had a normal tIgE level, ANA, anti-neutrophil cytoplasmic antibodies (ANCAs), anti-TPO, and anti-TG antibodies, but some features, including low levels of tIgA, might support the co-existence of autoimmune CSU and myasthenia gravis [50].

In an article published in 2023, the authors evaluated various serum biomarkers in a group of CSU patients. Serum tIgE and anti-TPO-IgG levels were analyzed with chemiluminescence immunoassays. The study showed that the presence of anti-TPO-IgG and especially low tIgE levels at baseline, along with ANA positivity, could be considered possible biomarkers pointing to an autoimmune background in patients with CSU [51].

In an additional retrospective study published in 2023, the authors investigated the possible association between anti-thyroid IgG autoantibodies, i.e., anti-TG-IgG or anti-TPO-IgG, in CSU patients with disease duration and specific clinical features. As the authors concluded, the anti-thyroid autoantibodies might not be suitable biomarkers to predict disease duration, severity, or response to antihistamines in CSU patients [52].

In a study published in 2022, the authors used an in-house-developed ELISA, which was based on the YH35324 reagent to capture the FcεRIα binding site of FcεRIα-specific antibodies, to detect anti-FcεRIα-IgG, anti-FcεRIα-IgM, and anti-FcεRIα-IgA autoantibodies in the sera of CSU patients and control individuals. The levels of IgG/IgA/IgM autoantibodies were presented as the ratio of YH35324 preincubated to mock-preincubated values. Reduced levels after the pre-incubation were calculated, and lower ratios represent higher levels of circulating autoantibodies. Higher anti-FcεRIα-IgG autoantibody levels were measured in the sera of CSU patients in comparison with those found in control individuals; moreover, higher levels of serum anti-FcεRIα-IgG/anti-FcεRIα-IgA were detected in the subgroup of patients with autoimmune CSU, i.e., those who were ASST and/or ANA positive. Serum levels of tIgE and fIgE were also measured with a standard and an in-house-developed immunoassay, respectively, and no significant correlation was found between tIgE/fIgE and the IgG/IgA/IgM autoantibodies [53].

In another article published in 2022, the researchers describe the in-house development and validation of an ELISA with a reliable cut-off point, which can be applied to the determination of anti-Fc*ε*RIα-IgG autoantibodies in CSU patients. According to the authors, the in-house-developed ELISA exhibited favorable analytical characteristics; using this assay, high levels of anti-Fc*ε*RIα-IgG autoantibodies could be detected in CSU patients, thus supporting previous studies indicating that these autoantibodies are associated with CSU pathogenesis [54].

In an interesting study published in 2022, the researchers developed, in-house, a new immunochemical method capable of detecting functional anti-FcεRIα-IgG and anti-IgE-IgG autoantibodies, one which can cross-link the FcεRIα molecules and IgE antibodies on the surface of mast cells and basophils in sera from CSU patients. Furthermore, they determined the anti-FcεRIα-IgG and anti-IgE-IgG autoantibodies in sera from autoimmune CSU patients using the new assay (AlphaCL, amplified luminescence proximity homogeneous assay by cross-linking) and a previously developed in-house ELISA. The results showed that the serum levels of anti-FcεRIα-IgG and anti-IgE-IgG autoantibodies in CSU patients and healthy individuals did not significantly differ when they were measured with the previously developed ELISA; on the contrary, a different pattern was observed when the AlphaCL was applied, i.e., using AlphaCL, anti-FcεRIα-IgG autoantibodies were detected in most patients with autoimmune CSU, but not in healthy subjects. According to the authors, the new immunochemical-type assay which can detect functional autoantibodies with experimentally demonstrated FcεRIα- and IgE-cross-linking ability, might serve the diagnosis of type IIb CSU in particular [55].

In a study published in 2021, the authors investigated a cohort of CU patients (87 with CSU, 33 with CIndU, and 18 with both forms), regarding patients’ response to omalizumab therapy. Among other parameters analyzed, serum tIgE was measured with a standard chemiluminescence immunoassay, while thyroid autoantibodies, such as serum anti-TPO-IgG and anti-TG-IgG, were determined with electrochemiluminescence immunoassays. The authors report that nonresponders had lower baseline tIgE levels; a significantly higher proportion of patients with low tIgE levels was found among nonresponders as compared with responders. Additionally, more nonresponders had elevated thyroid autoantibodies than responders. The median ratio of serum anti-TPO-IgG to serum tIgE was significantly higher in nonresponders than in responders. In addition, patients with higher baseline tIgE levels were more prone to rapid recurrence after discontinuation of omalizumab treatment [56].

In a retrospective study published in 2021, the authors investigated how high anti-TPO-IgG and low tIgE, independently and in combination, are linked to features of autoimmune CSU as well as to treatment responses. Demographic, clinical, and laboratory parameters and treatment responses were analyzed. Levels of anti-TPO-IgG and tIgE were measured in the local laboratories of all centers participating in the study, following assays with different cut-off values. Despite this difficulty, the findings suggest that the combination of high levels of anti-TPO-IgG and low levels of tIgE is a useful diagnostic marker for autoimmune CSU in everyday clinical practice [57].

In a study published in 2021, the authors investigated the genome-wide DNA methylation pattern in the whole blood of CSU patients. In this study, serum levels of tIgE, anti-TPO-IgG autoantibodies, and anti-TG-IgG autoantibodies were determined with standard immunoassays. The analysis showed that, of the 28 differentially methylated genes (DMGs) detected, *HLA*-*DPB2*, *HLA-DRB1*, *PPP2R5C*, and *LTF* were correlated with autoimmunity. CSU patients with anti-TPO-IgG and anti-TG-IgG positivity as well as elevated tIgE levels showed phenotype-specific differentially methylated positions (DMPs) [58].

In a 2020 study, the authors developed, in-house, an ELISA to determine the serum levels of autoantibodies against FcεRIα that belonged to one of the IgG, IgM, or IgA classes. The study showed that increased serum levels of anti-FcεRIα-IgM autoantibodies were often found in CSU patients and correlated with an autoimmune CSU profile; this is probably the first study reporting that CSU patients have IgM and IgA autoantibodies against FcεRIα, in addition to IgG autoantibodies. Total serum IgG, IgA, IgM, and IgE were also measured with routine laboratory assays, but no statistically significant findings were reported [59].

A multinational, multicenter study (the PURIST study) including 182 CSU patients was published in 2019. The parameters analyzed included serum levels of anti-FcεRI-IgG, anti-IgE-IgG, anti-TPO-IgG, and tIgE. Measurements of anti-FcεRI-IgG and anti-IgE-IgG were performed with in-house-developed ELISAs, while anti-TPO-IgG and tIgE levels were measured with standard immunoassays. A small proportion of the patients studied (8%, 15 patients), who fulfilled all three criteria of type IIb autoimmune CSU, exhibited significantly lower tIgE and higher anti-TPO-IgG levels [60].

Details concerning the measurement of specific IgG autoantibodies and/or tIgE levels in CSU patients, as described in the articles presented above, are summarized in Table 2.

### 3.3. Measurement of Specific IgE Autoantibodies, Specific IgG Autoantibodies and/or Total IgE Levels in CSU Patients

A number of articles referring to the immunochemical-assay-based measurement of specific IgE and IgG autoantibodies that may or may not co-exist in the samples from the same patient have been published in the recent literature. In a series of these articles, tIgE levels were also measured. The articles are detailed below.

In a recent study, the authors investigated how anti-TPO-IgE and anti-TPO-IgG autoantibodies may be associated with each other and correlated with CSU clinical characteristics, disease severity, and response to therapy. To achieve their objective, the authors measured anti-TPO-IgE and anti-TPO-IgG in the sera of 146 CSU patients and 30 control subjects, using an in-house-developed direct ELISA and a commercially available ELISA, respectively. Moreover, anti-TG-IgG, anti-FcεRI-IgG, anti-IgE-IgG, and anti-IL24-IgE were measured with a commercially available ELISA (anti-TG-IgG), in-house-developed direct ELISAs (anti-FcεRI-IgG, anti-IgE-IgG), and an in-house-developed indirect ELISA (anti-TG-IgG); tIgE was also measured with a commercially available immunoassay kit. The results showed that many CSU patients had autoantibodies against TPO, while most patients had either IgE or IgG autoantibodies, but not both. CSU patients showed different disease profiles depending on the presence of anti-TPO-IgE or anti-TPO-IgG autoantibodies; moreover, they exhibited differences in disease severity and response to therapy with antihistamines, depending on the positivity of each autoantibody. As the findings of the study revealed, CSU patients with negative anti-TPO-IgE showed greater disease activity and poorer response to therapy than those with positive anti-TPO-IgE. Mean tIgE was significantly higher in CSU patients than in control individuals; on the other hand, the anti-TPO-IgG/tIgE ratio, which is often associated with omalizumab response, showed no relation to response to antihistamine treatment in this study [62].

In another recent study, the researchers measured the levels of anti-FcεRI-IgE and anti-FcεRI-IgG autoantibodies along with anti-FcεRII-IgE and anti-FcεRII-IgG autoantibodies in CSU patients and control individuals; subsequently, they correlated the measured values with the response to omalizumab treatment. IgE/IgG-anti-FcεRI and IgE/IgG-anti-FcεRII autoantibodies were measured at baseline by a previously described sandwich ELISA. The study showed that significantly elevated anti-FcεRI-IgE autoantibody levels were measured in the CSU patients when compared with the control group; moreover, simultaneous anti-FcεRI-IgE and anti-FcεRI-IgG positivity was associated only with late response and non-response to omalizumab treatment [63].

In a separate study of 2023, the researchers assessed the proportions of CSU patients with different disease endotypes. More specifically, 111 CSU patients were subdivided into endotype I and endotype IIb. Endotype I was defined by detection of anti-TPO-IgE and anti-IL24-IgE autoantibodies with in-house-developed ELISAs; endotype IIb was defined by positive results of ASST and BAT biological assays along with positivity of in-house-developed ELISAs for anti-FcεRI-IgG and anti-IgE-IgG autoantibodies. Anti-TPO-IgG, ANA, and tIgE were also measured in all patients with routine laboratory assays. The results showed that endotypes I and IIb may co-exist in CSU patients, but these findings should be confirmed through further multicenter studies including larger patient cohorts; overall, further investigation is necessary to better understand disease pathophysiology and the exact role of specific IgE and/or IgG autoantibodies in CSU. Interestingly, tIgE levels were not in full agreement with those reported in previous relevant studies and this discrepancy has been attributed to the different assay methods used for determining tIgE [64].

In another recently published article, the authors investigated the risk factors for patients with SARS-CoV-2-vaccine-induced immediate allergy and CU. Among other factors, anti-IL24-IgE, anti-TPO-IgE, anti-FcεRI-IgG, anti-TPO-IgG, anti-thymidylate synthetase IgG autoantibodies (anti-TYMS-IgGs), and anti-thyroid hormone receptor alpha IgG autoantibodies (anti-THRA-IgGs) were investigated. Anti-IL24-IgE, anti-TPO-IgE and anti-FcεRI-IgG were assessed with in-house-developed ELISAs. The study showed that significantly increased levels of anti-IL24-IgE, anti-FcεRI-IgG, anti-TPO-IgG (in contrast to anti-TPO-IgE) as well as anti-TYMS-IgG and anti-THRA-IgG were found in SARS-CoV-2-vaccine-induced CU patients compared with SARS-CoV-2-vaccine-tolerant controls. Some of the SARS-CoV-2-vaccine-induced CU patients who did not respond to conventional therapy could be successfully treated with omalizumab. Interestingly, the authors reported that the autoantibodies found in the SARS-CoV-2-vaccine-induced CU patients were different from those detected in CSU patients (i.e., with disease not induced by SARS-CoV-2 vaccination) [65].

A 2022 meta-analysis of Chinese Han CSU patients showed that significantly higher prevalence of anti-TPO-IgE, anti-TPO-IgG, anti-TG-IgE, or anti-TG-IgG was observed in patients than in control individuals. Moreover, prevalence of anti-TPO-IgE and anti-TPO-IgG as well as of anti-TG-IgE and anti-TG-IgG autoantibodies was significantly correlated in CSU patients. Significantly lower tIgE levels were found in patients with anti-TPO-IgE and anti-TPO-IgG positivity; positive anti-TPO-IgE, positive anti-TPO-IgG and low tIgE levels were independent predictors of poor response to antihistamine therapy. Serum IgE and IgG autoantibodies and tIgE were measured using immunoassays [66].

In a 2021 study, the authors investigated anti-TPO-IgE in a group of patients with CSU and a group of patients with Hashimoto’s thyroiditis along with a control group of individuals without a history of Hashimoto’s thyroiditis and urticaria. Serum anti-TPO-IgE autoantibodies were measured with an in-house-developed site-directed IgE capture ELISA, after appropriate modifications. Moreover, serum anti-TPO-IgG was measured with a CLIA assay, while tIgE was also determined. The results showed that anti-TPO-IgE autoantibodies were detected in all participants of all groups, but no significant difference was observed among the three groups. Moreover, although higher tIgE and anti-TPO-IgE levels were detected in CSU patients who were anti-TPO-IgG positive, the differences observed were not significant. Overall, according to the authors, anti-TPO-IgE autoantibodies do not seem to play a primary pathogenic role, at least in many CSU patients [67].

In a study published in 2020, the authors evaluated, in parallel, IgE and IgG autoantibodies to TF; IgE and IgG autoantibodies to TG; IgE and IgG autoantibodies to FcεRI; and IgE and IgG autoantibodies to FcεRII in a group of CSU patients, and correlated positivity with response to omalizumab administration. More specifically, sera from patients and controls were analyzed for IgE and IgG autoantibodies at baseline with in-house-developed sandwich ELISAs. The results showed that autoantibodies of both IgE and IgG classes were detected in more than half of CSU patients. The autoimmune profile may possibly be correlated with and influence the clinical response to anti-IgE therapy with omalizumab, but further studies are necessary to draw a firmer conclusion. Total IgE was also measured with an immunoenzymatic method and higher tIgE levels were found in CSU patients in comparison with control individuals [68].

In a 2019 article, the authors measured the levels of anti-TPO-IgE and anti-TPO-IgG autoantibodies along with tIgE in patients with CSU, patients with autoimmune thyroid disease (ATD), and control individuals. Anti-TPO-IgE and anti-TPO-IgG levels were measured with in-house-developed ELISAs, while tIgE levels were assessed with a commercially available immunoassay. The results showed that anti-TPO-IgE autoantibodies were present in all groups and that their levels were highest in CSU patients, while anti-TPO-IgG levels were highest in ATD patients; moreover, the CSU patients exhibited higher prevalence of atopy and had higher tIgE levels than the ATD patients or the control individuals. According to the results of the ELISAs (as well as a biological assay), anti-TPO-IgE autoantibodies might not be a suitable specific biomarker for CSU, but they might participate in effector cell activation and disease progression, at least in some patients with CSU [39].

Details concerning measurement of specific IgE autoantibodies, IgG autoantibodies and/or total IgE levels in CSU patients are summarized in Table 3.

## 4. Challenges and Future Perspectives

**Figure 1:**
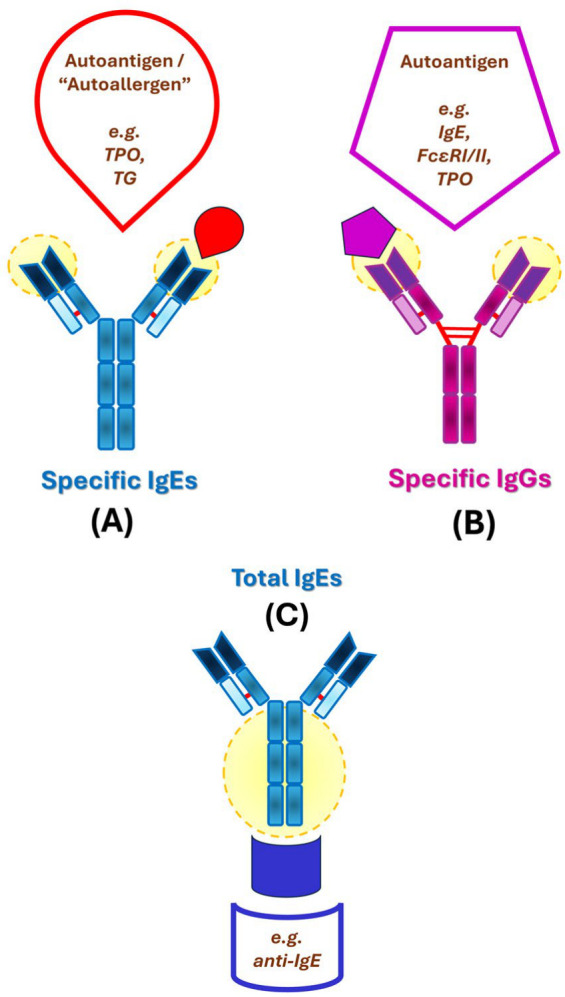
Schematic representation of the following three main CSU biomarkers. (**A**) Specific IgE autoantibodies against CSU-associated autoantigens/autoallergens. (**B**) Specific IgG autoantibodies against CSU-associated autoantigens. (**C**) Total IgE. Specific recombinant autoantigens (**A**,**B**) or secondary antibodies (**C**) are usually used in the immunochemical assays for determining IgE autoantibodies (**A**), IgG autoantibodies (**B**), or tIgE (**C**), as shown in the magnified views accompanying each represented immunoglobulin; moreover, the regions of the immunoglobulin molecules that are involved in the binding-based immunochemical assays are marked with dotted circles (**A**–**C**). Figure footnote: TPO: thyroid peroxidase; TG: thyroglobulin; IgE: immunoglobulin E; IgG: immunoglobulin G; FcεRI: high-affinity IgE receptor, FcεRII: low affinity IgE receptor.

IgE and/or IgG autoantibodies are considered key biomarkers in autoimmune CSU. The presence of such autoantibodies in CSU patients can be detected with a series of functionality-based biological assays, while their levels can be quantified with various binding-based immunochemical assays, predominantly ELISAs. As a matter of fact, specific IgE autoantibodies (especially anti-TPO-IgE and to a lesser extent anti-TG-IgE), specific IgG autoantibodies (such as anti-IgE-IgG and anti-FcεRI-IgG, or anti-TPO-IgG) as well as tIgE levels, alone or in various combinations, are considered predominant biomarkers and may serve CSU diagnosis and endotyping and the prognosis and/or prediction of treatment response. As already mentioned, specific IgE autoantibodies, specific IgG autoantibodies, and tIgE can be determined in patients’ sera with binding-based immunochemical assays, in which specific regions of the immunoglobulin molecule are recognized by specific agents, such as recombinant autoantigens or secondary antibodies (Figure 1).

Many of the immunochemical assays for determining CSU-associated autoantibodies have been in-house developed, as shown in Table 1, Table 2 and Table 3, while there is a shortage of widely available commercial assay kits that can be used by different research groups worldwide or, above all, in a clinical setting, which may lead to inconsistencies among the results presented in different reports. This is especially true for type I CSU (autoallergic), which has been correlated with the presence of IgE autoantibodies such as anti-TPO-IgE or anti-IL24-IgE. In contrast to classical allergy, in which several well-validated and approved immunochemical assays can be applied worldwide for the detection of allergen-specific IgEs, assays for CSU-associated IgE autoantibodies have been in-house developed by research groups and used at specific laboratories, e.g., some Urticaria Centers of Reference and Excellence, but they are not available for routine clinical use [22,23]. As reported, this is probably the reason why such different prevalence rates can be found in the literature for CSU-associated autoantibodies, e.g., 0–100% for anti-TPO-IgE [23]. More specifically, such variations can be attributed, at least in part, to differences in the assay format and/or reagents of the immunochemical methods applied to determine specific IgE autoantibodies, such as anti-TPO-IgE [22,32,69]. Overall, predominantly in-house developed protocols have been and are still being used [70] for investigating the relationship between specific IgE autoantibodies and CSU, which implies that there is still a risk of highly variable outcomes.

Similarly, immunoassays for anti-IgE-IgG and anti-FcεRI-IgG, which have been correlated with type IIb CSU, are mostly in-house developed by individual research groups and thus not widely available for routine clinical practice; moreover, well-validated and standardized immunoassays for determining anti-IgE and anti-FcεRI autoantibodies of the IgM or the IgA class are generally not available [22] and this has contributed to our incomplete knowledge concerning the role of specific IgM and/or IgA autoantibodies in type IIb CSU [16]. In addition, the existing immunoassays for determining CSU-associated IgG autoantibodies do not usually discriminate among IgG subclasses, some of which are present in CSU patients but are not functional, and this might explain some discrepancies observed among the results obtained with functionality-based biological assays and binding-based immunochemical ones [18].

As already mentioned, another useful diagnostic marker for type IIb CSU that can be more easily applied in clinical practice is high anti-TPO-IgG combined with low tIgE levels. However, the measurement of anti-TPO-IgG autoantibodies is not yet globally standardized, and this may explain the variability in the results obtained across different studies [62], while well-established “cut-off” values of tIgE have yet to be adopted globally; overall, it is generally well accepted that there is a need to better define the predictive values, sensitivity, and specificity of using anti-TPO-IgG and serum tIgE as biomarkers in CSU [25].

Mean serum tIgE levels have often been studied in CSU patients and have been proposed as a biomarker of type I or type IIb CSU [16,18,22,26], or as a factor predicting the clinical outcomes of various CSU therapeutics [56,71,72]; however, the results obtained across different studies are often contradictory [73]. As suggested, the use of the same World Health Organization (WHO) international reference preparation for human IgE minimizes significant systematic errors and facilitates the comparison of serum tIgE levels obtained with different quantitative assays [57]. Nevertheless, despite the use of a common WHO reference material for tIgE assay calibration, significant differences in quantitation between two FDA-cleared test systems are reported elsewhere [74]. Moreover, even when well-characterized, commercially available immunoassay kits are used for quantifying tIgE levels, the cut-off values of each of the immunoassay kits that are used to discriminate among “low”, “normal”, and “high” should be further validated, normalized, and standardized [57], and further efforts must be made to harmonize and improve the reporting of IgE reference intervals [75]. In a few cases, in addition to tIgE and fIgE levels, IgE not bound to specific receptors or therapeutically administered anti-IgE antibodies have been measured, especially when CSU patients receiving treatment with anti-IgE monoclonal antibodies, such as omalizumab, have been monitored [40,53]. Standardization of assays measuring fIgE in patients’ sera should also be addressed; the same requirement also applies to assays used for determining tIgA and tIgM levels in patients with CSU.

The recent finding that a subpopulation of CSU patients exhibits features of both type I and type IIb CSU has highlighted the need to further study the clinical implications of dual IgE and IgG autoantibodies [19]. Moreover, as reported, the overlap of IgE and IgG autoantibodies may affect the response to currently available and future envisaged therapeutics [22]; for instance, the coexistence of IgG autoantibodies with IgE autoantibodies has been associated with a late or a lack of response to omalizumab [25]. The coexistence of IgE and IgG autoantibodies, especially when they are targeting the same autoantigen, should be carefully investigated using highly specific and reliable immunochemical assays for each autoantibody. Further investigation of a putative relationship between the autoantibody titers, as assessed by such immunoassays, and disease activity would be of interest and might prove of predictive value.

It should be noted that, to date, the immunochemical assays for determining CSU-relevant autoantibodies have been predominantly used in research studies or as supplementary methods to help with diagnosis/prognosis and/or prediction of response to specific therapeutics [26]. In this respect, they differ from immunochemical methods, e.g., direct immunofluorescence microscopy or indirect immunofluorescence, which are currently used as well-established diagnostic tools for other autoimmune skin diseases, such as mucous membrane pemphigoid [76].

Overall, there is probably a need to develop well-standardized and widely available assays for CSU-associated IgE and IgG autoantibodies against known autoantigens; moreover, it is necessary to better standardize the immunoassay methodology used so far for determining tIgE in CSU patients. In addition, further investigations should focus on discovering new autoantigens/autoallergens and autoantibodies associated with CSU, for which specific immunochemical assays will consequently be developed [21,22].

## 5. Conclusions

CSU is a skin disease manifested by almost daily wheals or angioedema that last for more than six weeks. CSU has been reported to affect 1% to 2% of the general population; thus, it may influence the quality of life of a great number of human individuals and increase health care costs. A great proportion of CSU cases are associated with autoimmune mechanisms. To date, immunochemical assays for determining CSU-associated specific IgE and IgG autoantibodies, along with assays for determining tIgE (or tIgE/fIgE), have been an invaluable laboratory tool, one which has supported research into autoimmune endotypes of CSU over the last several years. Further validation of the existing assays, along with the development of reliable assays for novel autoantigens and autoantibodies, will unequivocally help us gain deeper and more thorough knowledge concerning CSU pathogenesis and will ultimately facilitate clinical translation.

## Figures and Tables

**Table 1:** Specific IgE autoantibodies measured, mostly along with tIgE levels, in sera of CSU patients.

Specific IgE: Relevant Autoantigen	Specific IgE: Immunochemical Assay Used for Analysis	tIgE: Yes/No	tIgE: Immunochemical Assay Used for Analysis	Number of Patients	Disease	Ref.
Tg2	Capture ELISA (in-house developed)	Yes * (additionally: tIgA, tIgG, tIgM)	---	160	CSU	[33]
TPO	Immunochemiluminescence assay	No	N/A	217	CSU, Atopic dermatitis, Atopic rhinitis	[34]
TG	Immunochemiluminescence assay					
EPX	ELISA (in-house developed)	Yes	Fluorenzyme immunoassay	101	CSU, Atopic dermatitis	[36]
ECP	ELISA (in-house developed)					
TPO	ELISA (in-house developed)					
TPO	ELISA (in-house developed)	Yes * (additionally: tIgA, tIgG, tIgM)	---	606	CSU	[37]
TPO	ELISA (in-house developed (as described in [39]))	Yes	---	100	CSU	[38]

Abbreviations: CSU: chronic spontaneous urticaria; ECP: eosinophil cationic protein; ELISA: enzyme-linked immunosorbent assay; EPX: eosinophil peroxidase; IgE: immunoglobulin E; N/A: not applicable; TG: thyroglobulin; Tg2: tissue transglutaminase 2; tIgA: total immunoglobulin A; tIgE: total immunoglobulin E; tIgG: total immunoglobulin G; tIgM: total immunoglobulin M; TPO: thyroid peroxidase. *: In addition to tIgE, tIgA, tIgG and tIgM were also measured. ---: No exact information is provided in the corresponding article.

**Table 2:** Specific IgG autoantibodies measured, mostly along with tIgE levels, in CSU patients.

Specific IgG: Relevant Autoantigen	Specific IgG: Immunochemical Assay Used for Analysis	tIgE: Yes/No	tIgE: Immunochemical Assay Used for Analysis	Biological Sample	Number of Patients	Ref.
IgE	ELISA	Yes ^&^	ELISAs	Sera from CSU patients prior to omalizumab treatment	61	[40]
FcεRIα	ELISA					
ANA	Indirect immunofluorescence biochip kit (microscopy assessment)	Yes	---	Sera from CSU patients	60	[41]
TPO	CLIA	No	N/A	Sera from CSU patients	30	[42]
TPO	Sandwich ECLIA	Yes	Sandwich ECLIA	Sera from CSU patients under omalizumab treatment	52	[43]
IgE	ELISA (in-house developed)	No	N/A	Sera from CSU patients	49	[44]
FcεRI	ELISA (in-house developed)					
TPO	EFIA	Yes	EFIA	Sera from CSU patients under omalizumab treatment	296	[47]
HSP10	Immune dot-blot assay (in-house developed)	Yes	ImmunoCAP fluoroenzyme immunoassay system	Sera from CSU patients	86	[49]
TPO	CLIA	Yes	CLIA	Sera from CSU patients	377	[51]
TPO	---	No	N/A	Blood from CSU patients	147	[52]
TG	---					
FcεRIα	ELISA * (in-house developed)	Yes ^&^ tIgE	ImmunoCAP fluoroenzyme immunoassay system	Sera from CSU patients	88	[53]
		fIgE	ELISA (in-house developed, as described in [61])			
FcεRIα	ELISA (in-house developed)	No	N/A	Sera from CSU patients	233	[54]
IgE	ELISA (in-house developed as described in [59], with slight modifications),	No	N/A	Sera from CSU patients	14	[55]
	and					
	Amplified luminescence proximity homogeneous assay with cross-linking, AlphaCL (in-house developed)					
FcεRIα	ELISA (in-house developed as described in [59], with slight modifications),					
	and					
	Amplified luminescence proximity homogeneous assay with cross-linking, AlphaCL (in-house developed)					
TG	ECLIA	Yes	CLIA	Sera from CSU and CIndU patients under omalizumab treatment	138	[56]
TPO	ECLIA					
TPO	Commercially available assay kits	Yes	Commercially available assay kits	Sera from CSU patients	1120	[57]
TPO	Immunoassay	Yes	ELISA	Sera from CSU patients	95	[58]
TG	Immunoassay					
FcεRIα	ELISAs * (in-house developed)	Yes **	Routine laboratory measurement **	Sera from CSU patients	35	[59]
FcεRIα	ELISA (in-house developed)	Yes	ImmunoCAP fluoroenzyme immunoassay system or nephelometric	Sera from CSU patients	182	[60]
IgE	ELISA (in-house developed)					
TPO	Immunoassay					

Abbreviations: ANA: anti-nuclear autoantibody; CIndU: chronic inducible urticaria; CLIA: chemiluminescence immunoassay; ECLIA: electrochemiluminescence immunoassay; EFIA: enzyme-fluoroimmunoassay; FcεRI: high-affinity IgE receptor; FcεRIα: extracellular α-chain of high-affinity IgE receptor; FcεRII: low-affinity IgE receptor; fIgE: free immunoglobulin E; HSP10: heat-shock protein 10; TG: thyroglobulin; tIgE: total immunoglobulin E; TPO: thyroid peroxidase. *: ELISAs for specific IgM and IgA autoantibodies were also developed in-house and applied to the measurement of anti-FcεRIα autoantibodies of the IgM and IgA classes. **: In addition to total IgE, total IgG, total IgM, and total IgA were also measured by “routine laboratory methods”, which was not further defined. ^&^: Both tIgE and fIgE were measured; fIgE was measured using the so-called YH35324 reagent, which could capture the FcεRIα binding site of FcεRIα-specific antibodies. ---: Further details are not provided.

**Table 3:** Specific IgE and specific IgG autoantibodies measured, mostly along with tIgE levels, in CSU patients.

Specific IgE: Relevant Autoantigen	Specific IgE: Immunochemical Assay Used for Analysis	Specific IgG: Relevant Autoantigen	Specific IgG: Immunochemical Assay Used for Analysis	tIgE: Yes/No	tIgE: Immunochemical Assay Used for Analysis	Biological Sample	Number of Patients	Ref.
TPO	Direct ELISA (in-house developed, as described in [69], with modifications)	TPO	ELISA	Yes	ImmunoCAP fluoroenzyme immunoassay system	Sera from CSU patients	146	[62]
		TG	ELISA					
IL24	Site-directed ELISA (in-house developed, as described in [64])	IgE	Direct ELISA (in-house developed, as described in [64])					
		FcεRI	Direct ELISA (in-house developed, as described in [64])					
FcεRI	Sandwich ELISA (in-house developed, as described in [68])	FcεRI	Sandwich ELISA (in-house developed, as described in [68])	No	N/A	Sera from CSU patients under omalizumab treatment	18	[63]
FcεRII	Sandwich ELISA (in-house developed, as described in [68])	FcεRII	Sandwich ELISA (in-house developed, as described in [68])					
IL24	ELISA (in-house developed)	IgE	ELISA (in-house developed)	Yes	Phadia ImmunoCAP System	Sera from CSU patients	111	[64]
TPO	ELISA (in-house developed)	FcεRI	ELISA (in-house developed)					
		TPO	--- (“routine laboratory measurement”)					
IL24	ELISA (in-house developed)	FcεRI	ELISA (in-house developed, as described in [60])	Yes	---	Sera from CU patients after SARS-CoV-2 vaccination	129	[65]
TPO	ELISA (in-house developed)	TPO	---					
		TYMS	---					
		THRA	---					
TPO	ELISA	TPO	Chemiluminescence microparticle immunoassay	Yes	ELISA	Sera from CSU patients	1100	[66]
TG	ELISA	TG	Chemiluminescence microparticle immunoassay					
TPO	Site-directed capture ELISA (in-house developed, as described in [32], with modifications)	TPO	Chemiluminescence microparticle immunoassay	Yes	---	Sera from CSU and Hashimoto’s thyroiditis patients	117	[67]
FcεRI	Sandwich ELISA (in-house developed)	FcεRI	Sandwich ELISA (in-house developed)	Yes	Enzyme immunoassay	Sera from CSU patients under omalizumab treatment	20	[68]
FcεRII	Sandwich ELISA (in-house developed)	FcεRII	Sandwich ELISA (in-house developed)					
TF	Sandwich ELISA (in-house developed)	TF	Sandwich ELISA (in-house developed)					
TG	Sandwich ELISA (in-house developed)	TG	Sandwich ELISA (in-house developed)					
TPO	ELISA (in-house developed)	TPO	ELISA (in-house developed)	Yes	ImmunoCAP fluoroenzyme immunoassay system	Sera from CSU and ATD patients	130	[39]

Abbreviations: ATD: autoimmune thyroid disease; FcεRI: high-affinity IgE receptor; FcεRII: low-affinity IgE receptor; IL24: interleukin 24; tIgE: total immunoglobulin E; TF: tissue factor; TG: thyroglobulin; THRA: thyroid hormone receptor alpha; TPO: thyroid peroxidase; TYMS: thymidylate synthetase. ---: No exact information is provided in the corresponding article.

## Data Availability

No new data were created or analyzed in this study.

## Abbreviations

The following abbreviations are used in this manuscript:

AD	Autoimmune disease
ANA	Anti-nuclear autoantibodies
ANCA	Anti-neutrophil cytoplasmic antibodies
ASST	Autologous serum skin test
ATD	Autoimmune thyroid disease
BAT	Basophil activation test
BHRA	Basophil histamine release assay
BLR	Basophil to lymphocyte ratio
CaU	Chronic autoimmune urticaria
CLIA	Chemiluminescence immunoassay
CIndU	Chronic inducible urticaria
CSU	Chronic spontaneous urticaria
CU	Chronic urticaria
ECLIA	Electrochemiluminescence immunoassay
EFIA	Enzymatic fluoroimmunoassay
ELISA	Enzyme-linked immunosorbent assay
ELR	Eosinophil to lymphocyte ratio
EPX	Eosinophil peroxidase
FcεRI	High-affinity IgE receptor
FcεRIα	Extracellular alpha chain of FcεRI
FcεRII	Low-affinity IgE receptor
FDA	Food and Drug Administration
fIgE	Free immunoglobulin E
HSP10	Heat shock protein 10
IgA	Immunoglobulin A
IgE	Immunoglobulin E
IgG	Immunoglobulin G
IgM	Immunoglobulin M
IIF	Indirect immunofluorescence assay
IL24	Interleukin 24
MG	Myasthenia gravis
NLR	Neutrophil to lymphocyte ratio
PLR	Platelet to lymphocyte ratio
TF	Tissue factor
TG	Thyroglobulin
Tg2	Tissue transglutaminase 2
THRA	Thyroid hormone receptor alpha
tIgA	Total immunoglobulin A
tIgE	Total immunoglobulin E
tIgG	Total immunoglobulin G
tIgM	Total immunoglobulin M
TPO	Thyroid peroxidase
TYMS	Thymidylate synthetase
WHO	World Health Organization
